# Natural parsley oil as a green and safe inhibitor for corrosion of X80 carbon steel in 0.5 M H_2_SO_4_ solution: a chemical, electrochemical, DFT and MC simulation approach

**DOI:** 10.1039/d1ra08855f

**Published:** 2022-01-21

**Authors:** M. Abdallah, K. A. Soliman, B. A. Al Jahdaly, Jabir H. Al-Fahemi, H. Hawsawi, H. M. Altass, M. S. Motawea, Salih S. Al-Juaid

**Affiliations:** Chem. Depart. Faculty of Applied Sciences, Umm Al-Qura University Makkah Saudi Arabia metwally555@yahoo.com; Chem. Depart. Faculty of Science, Benha University Benha Egypt; University College of Alwajh, Tabuk University Tabuk Saudi Arabia; Chem. Depart. Faculty of Science, Tabuk University Tabuk Saudi Arabia; Chem. Depart., Faculty of Science, King Abdulaziz University Jeddah Saudi Arabia

## Abstract

This work focuses on the use of natural parsley oil as a safe, eco-friendly and cost-effective inhibitor for dissolution of X80 carbon steel (X80CS) in 0.5 M H_2_SO_4_ solution. Electrochemical and chemical measurements and theoretical studies were utilized to determine the inhibitory vigor of parsley oil. The inhibition efficacy increases with an increase in the parsley oil concentration and a decrease in temperature. It reached 95.68% at 450 ppm of parsley oil. The inhibition process is explained by spontaneous adsorption of the oil on the X80CS. Adsorption is described by the Langmuir isotherm model. The polarization data demonstrate that parsley oil is categorized as a mixed inhibitor with a dominant control of the cathodic reaction. Parsley oil inhibits the pitting corrosion of X80CS in the presence of NaCl solution by moving the pitting potential to a more positive mode indicating protection against pitting attack. The thermodynamic parameters for activation and adsorption were computed and interpreted. The four chemical components in natural parsley oil were examined using density functional theory (DFT). Monte Carlo (MC) simulation was performed to study the adsorption of parsley oil on the X80CS surface. The outcomes confirmed that the Apiole molecule is the most effective in the inhibition process.

## Abstract 2

This work focuses on the use of natural parsley oil as a safe, eco-friendly and cost-effective inhibitor for dissolution of X80 carbon steel (X80CS) in 0.5 M H_2_SO_4_ solution.

## Graphical abstract

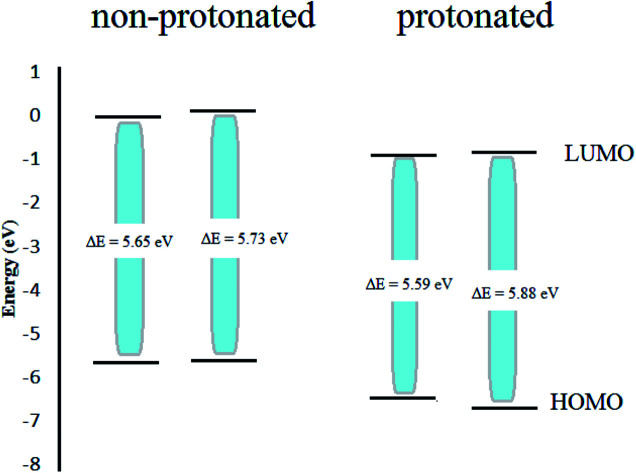

## 1. Introduction

The corrosion and inhibition of X80 carbon steel (X80CS) in acidic solutions is an important topic to study because of the use of this type of steel in many vital and important industries. Usually we use 0.5 M H_2_SO_4_ solutions for the pickling and chemical cleaning of X80 carbon steel but unfortunately, the acid corrodes the steel. There are several ways to protect carbon steel from corrosion hazards and one of these methods is to apply corrosion inhibitors to reduce the rate of corrosion and protect the X80CS from the dangers of corrosion.^1,2^ Most of these inhibitors are organic compounds that contain hetro nitrogen, oxygen or sulfur atoms or a mixture of two or three of these atoms and aromatic rings. The compounds give high protection against the corrosion of steel in acidic solution.^3–15^ Its inhibitory impact is attributed to its absorption on the surface of the steel. The adsorption efficacy relies on the chemical composition of the additive and the nature of the metals or alloys used, temperature, pH of the acid used and other factors. Most of these compounds gives high inhibition efficacy but the disadvantages are that they are expensive, uneconomical, toxic, and cause harm to the environment and human health.

To overcome the problems arising from organic compounds, scientists have turned to using some vegetable and essential oils as corrosion inhibitors for carbon steel in acidic solutions. These natural oils are safe, environmentally friendly, harmless to humans, and economically viable because their price is low when compared to other compounds. They also characterized by containing effective compounds and groups that have a high adsorption capacity on the surface of the steel and give a high inhibition efficiency.^16–25^

The strategic purpose of this work is to explore the anticorrosion property of natural oil such as parsley oil towards the corrosion of X80 carbon steel (X80CS) in a 0.5 M H_2_SO_4_ solutions. Chemical technology such as weight reduction (WR) and electrochemical technologies such as galvanoststic polarization (GAP), potentidynamic anodic polarization curves (PDAP) and electrochemical impedance spectroscopy (EIS) technologies were utilized to determine the inhibition efficiency of this oil. Also, computations studies are also applied to link between experimental and theoretical parameters. The study of the four component found in parsley oil using density functional theory (DFT) to compare and predict the most molecule of the four component is effective in inhibition process through some quantum descriptors. The Monte Carlo simulation is explored for study the adsorption of parsley oil on the X80CS surface.

## 2. Experimental

### 2.1. Materials

X80 carbon steel (X80 CS) sheet or rod utilized in this work has the chemical composition (weight%): C = 0.068, S = 0.024, P = 0.007, N = 0.005, Si = 0.192, Mn = 1.824, Mo = 0.015, Ni = 0.172, Al = 0.029, Ti = 0.014, Nb = 0.058, V = 0.002, Cu = 0.024, Cr 0.025, and the remainder being iron. All chemicals used were supplied from Sigma-Aldrich.

### 2.2. Techniques

For WR technique, X80 CS sheets with dimensions of 1.6 cm × 3.4 cm × 0.15 cm were used. For the GAP, PDAP and EIS techniques, the X80CS electrode engrossed in Araldite with an uncovered surface area of 0.36 cm^2^. Prior any experiment the X80CS slab or rod is sanded with some sand papers (range between 200 and 1400). After sanding it is washed with distilled water and degreased with acetone and lastly dried.

WR measurements were performed as previously described.^26^ Experiments were performed at diverse temperatures ranging from 298 K to 328 K ± 0.05 °C using an air thermostat. For GAP, PDAP and EIS measurements. Three electrodes were inserted into the cell used in this work, X80CS rod as the working electrode, Pt foil was utilized as an auxiliary electrode and a saturated calomel electrode (SCE) as reference electrode. The electrode potential was measured against SCE and it was allowed to stabilize for 30 min before starting the measurements. GAP studies were done using a PS remote potentiostat at scan rate 5.0 mV sec^−1^ with PS6 software used for calculation of some corrosion parameter such as corrosion potential (*E*_corr_), corrosion current density (*I*_corr_) and anodic (*β*_a_) cathodic (*β*_c_) Tafel slopes. These parameters were determined from the intersection of the anodic and cathodic Tafel lines.

PDAP measurements was performed utilizing a Wenking potentiostat type POS73 and an X–Y recorder, type advanced, HR2000. The experiments were done at a scanning rate 0.5 mV sec^−1^. Pitting potential (*E*_pitt._) is determined from PDAP technique. EIS was carried out using the frequency range of 100 kHz to 10 mHz during an amplitude of 5 mV peaks using a computer-controlled device (Auto Lab 30, Metrohm).

### 2.3. Inhibitor

In this study, parsley oil was used as a corrosion inhibitor for steel. Inhibitor stock solution (1000 ppm) was prepared by was prepared by dissolving it in a 30% by volume of ethyl alcohol in water and the required concentration of oil was prepared by dilution using bi-distilled water. The amount of ethyl alcohol in all aqueous solution in the presence and the absence of the investigated nutmeg oil were kept constant to remove the effect of the ethyl alcohol on the inhibition efficiency. The main chemical components of natural parsley oil (%) used as inhibitors in this study are given in Table 1.

**Table 1:** The chemical components of natural parsley oil

Myristicin (36.2%)	Apiole (21.0%)	α-Pinene (15.5%)	β-Pinene (10.4%)
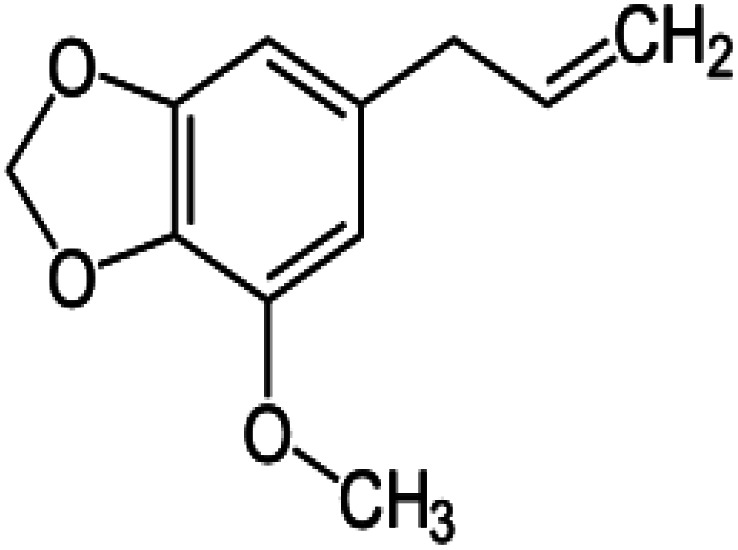	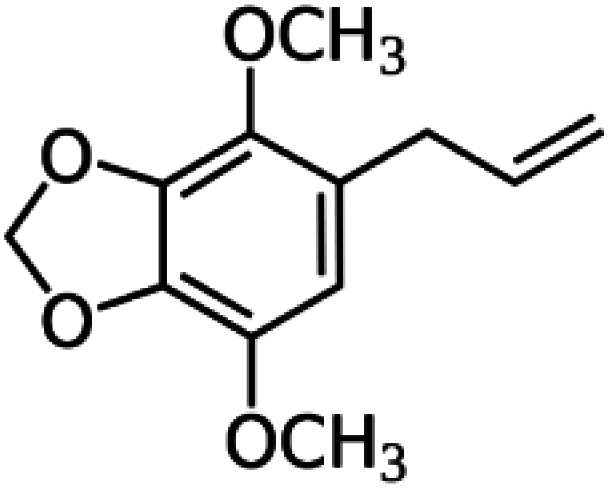	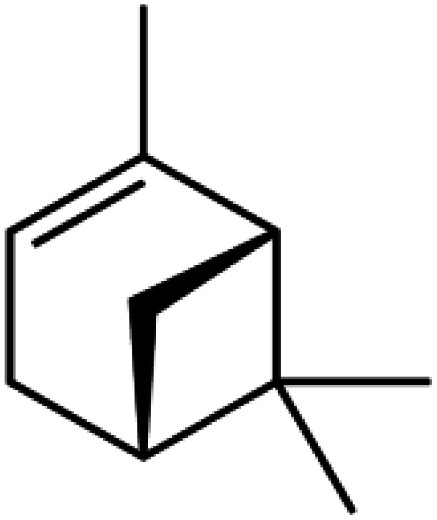	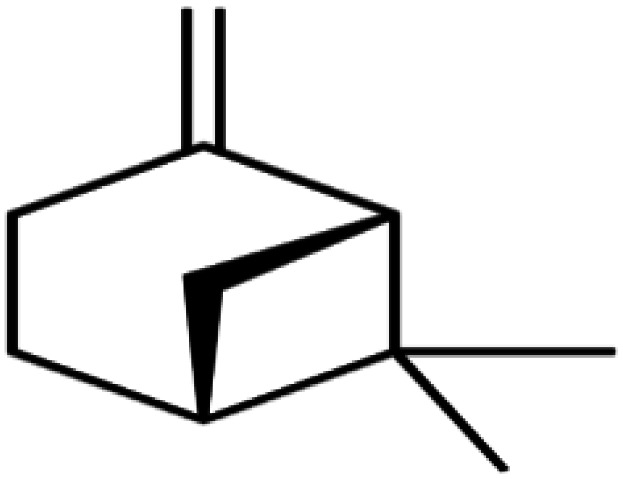

### 2.4. Computational details

The Apiole and Myristicin molecules present in all-natural parsley oil are optimized for both gaseous and aqueous phase. The optimization was performed using density functional theory ( DFT) with B3LYP level of theory^27–29^ and 6-13G (d, p) basis set. In previous study, both α-pinene and β-pinene compounds were optimized at the same basis set and level of theory.^16^ The conductor-like polarizable continuum model (CPCM) was used for solvation. All calculations were carried by Gaussian 09 code.^30^ Some quantum descriptors such HOMO, LUMO, energy gap (Δ*E*), and dipole moment were determined.

The hardness (*η*), softness (*σ*), and the fraction of electron transferred (Δ*N*) was also calculated by the following equations:1
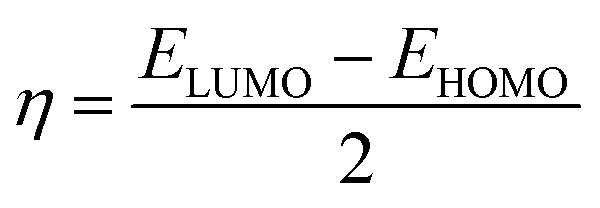
2
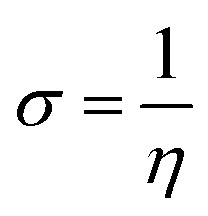
3
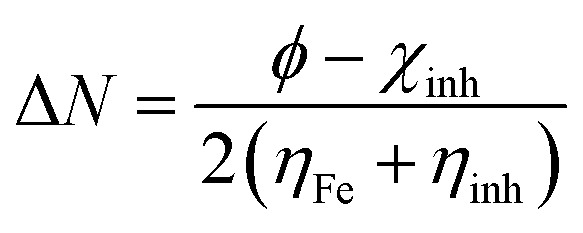
where, *ϕ*, *χ*_inh_, *η*_Fe_, and *η*_inh_ are the work function (4.82 eV), the electronegativity of inhibitor, hardness of Fe(110) surface (0 eV), and the hardness of the inhibitor.

### 2.5. Monte Carlo (MC) simulation

The adsorption of the Apiole and Myristicin molecules on the Fe (110) surface was investigated by the adsorption locator module. The Fe (110) surface composed of four layers and a supercell 5 × 5 were constructed with a vacuum 15 Å above the surface. The condensed-phase optimized molecular potentials for atomistic simulation studies (COMPASS) were used for the adsorption process. For the summation method, the Ewald method was set for an electrostatic and atom-based method for van der Waals. The adsorption energy of the inhibitor was computed by the subsequent equation:

4 *E*_ads_ = *E*_complex_ − *E*_Fe_ − *E*_inh_

where, *E*_complex_, *E*_Fe_, and *E*_inh_ are the total energies of inhibitor molecule on Fe (110) surface, the energy of Fe(110) surface, and the energy of inhibitor molecule respectively.

## 3. Results and discussion

### 3.1. GAP studies

**Fig. 1:**
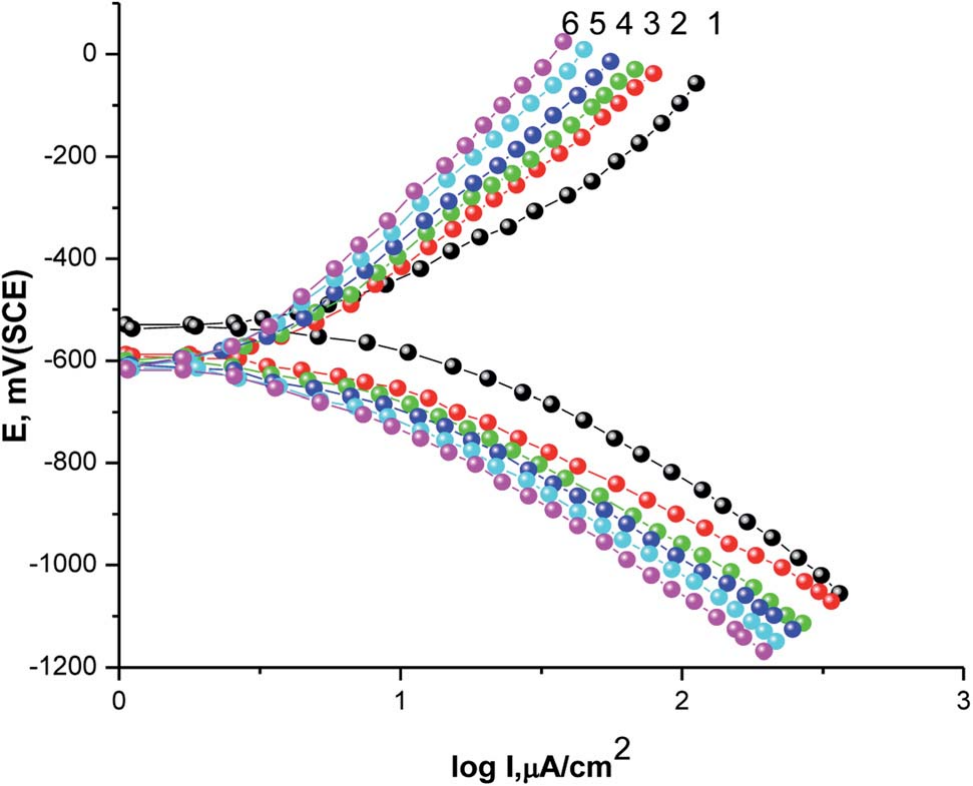
GAP curves of X80 CS in 0.5 M H_2_SO_4_ as well as containing certain concentricity of parsley oil. (1) 0.00 (2) 50 (3) 150 (4) 250 (5) 350 (6) 450 ppm.

The GAP curves for X80 CS corrosion in 0.5 M H_2_SO_4_ solutions in the absence and presence of various concentrations of parsley oil at 25 °C are represented Fig. 1. The corrosion data deduced from these curves such as *β*_a_, *β*_c_, *E*_corr_, *I*_corr_ and *P*% are registered in Table 2.

**Table 2:** GP parameters for X80 CS corrosion in 0.5 M H_2_SO_4_ alone and with various doses of parsley oil at 298 K

Oil. conc. (ppm)	−*β*_c_ (mV dec^−1^)	*β*_a_ (mV dec^−1^)	−*E*_corr_, mV (SCE)	*I*_corr_ mA cm^−2^	*P*_(PDP)_%
0.00	90	77	535	0.880	—
50	119	116	590	0.245	72.16
150	134	125	600	0.178	79.77
250	144	138	605	0.126	85.68
350	152	146	609	0.082	90.68
450	162	158	610	0.038	95.68

The inhibition efficacy (*P*_(PDP)_%) was computed from the values of *I*_corr_. utilizing the next equation;5
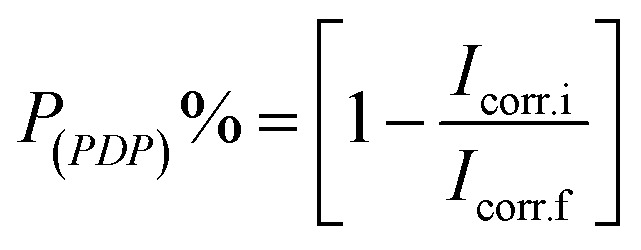
where, *I*_corr.f_ and *I*_corr.i_ are the corrosion current densities present in the free 0.5 M H_2_SO_4_ solutions and with the presence of different concentrations of parsley oil.

Obviously, with the gradual addition of different concentrations parsley oil concentrate to the corrosive 0.5 M H_2_SO_4_ solution hampered the anodic dissolution reaction of the steel and cathodic H_2_ evolution. The values of *β*_a_ and *β*_c_ are altered due to the adsorbing of parsley oil on the surface of the X80 CS by forming a covering layer on it.^31^ Similarly, it was found that, *E*_corr._ has turned to more negative potential in the occurrence of the of the parsley oil, which demonstrates that the adsorption mechanism on the X80 CS surface subject to the adsorption mode which should impede both the anodic and cathodic reactions. The maximum shift in the *E*_corr._ was 75 mV. According to the adsorption mode, this oil is a mixed inhibitor with dominant control of the cathodic reaction in which the value of *E*_corr_ displacement was less than 85 mV and the resulting shift indicates a nobler trend.^32^ The values of *I*_corr._ decreases with increasing the concentration of parsley oil and thus *P*% values increase. This confirms the vigor of its adsorption on the steel surface and the inhibiting power of this oil.

### 3.2. EIS measurements

**Fig. 2:**
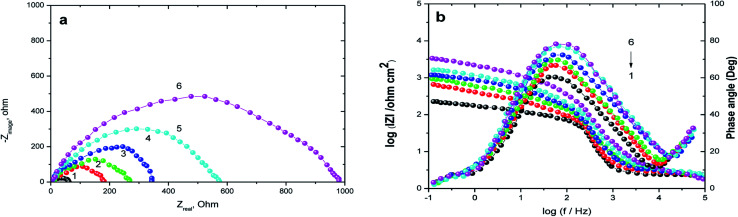
Nyquist (a) and Bode plots (b)of X80 CS corrosion in 0.5 M H_2_SO_4_ alone and in the presence of certain concentrations of parsley oil at 298 K. (1) 0.00 (2) 50 (3) 150 (4) 250 (5) 350 (6) 450 ppm.

**Fig. 3:**
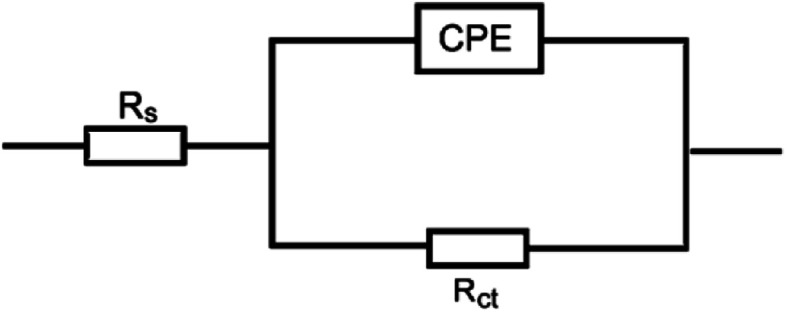
Electrochemical equivalent circuit utilized to fit the EIS parameters.

EIS technique as a non-destructive and an important tool for elucidation of the details of corrosion and corrosion inhibition has been reported in most of literature.^33^Fig. 2(a and b) represent the Nyquist and Bode plots for the corrosion of X80 CS in 0.5 M H_2_SO_4_ solution in the absence and presence of various concentrations of parsley oil at 298 K, respectively. It is observed from Fig. 2 a that there is a single semicircular capacitive loop in the inhibitor free solution denotes a single charge transfer process which controls the X80 CS corrosion process in this medium.^34^ The unaffected shape of this loop in the presence of parsley oil with different concentrations assist that this type of corrosion processes is under activation-controlled nature. As noted before this shape of the depressed semicircles in Nyquist curves are due to the irregularity of the surface formed at the steel–solution interface leaving inhomogeneity and roughness.^35^ The model used in EIS analysis is a constant-phase element (CPE) as shown in (Fig. 3).

The *P*_(EIS)_% were determined according to the next equation:6
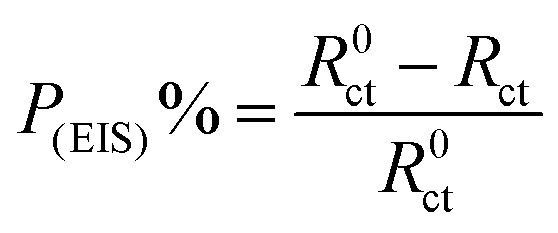
Where*, R*_ct_ and *R*^0^_ct_ are the charge transfer resistances in the existence and absence of parsley oil, respectively. The electrochemical parameters such as: *R*_ct_, *C*_dl_ and *P*_(EIS)_% are recorded in Table 3. The main point observed in Fig. 3, an obvious increase of semicircles diameters with adding the parsley oil which actually assist on the protective action of the used oil.^35^ This behavior explained by the increased values of *R*_ct_ (Table 3) suggesting the construction of a protective film on the X80 CS-electrolyte interface which is remarkably supported by the observed decrease in the *C*_dl_ values especially at higher concentricity of parsley oil. This reveals that the adsorption of the four main component of the parsley oil on the surface of the corrosive X80 CS instead of initially adsorbed water molecules.^36^

**Table 3:** EIS parameters for X80 CS corrosion in 0.5 M H_2_SO_4_ solution alone and with various concentrations of parsley oil at 298 K

Oil. conc. (ppm)	*R*_s_ (Ω cm^2^)	*R*_ct_ (Ω cm^2^)	*C*_dl_ (μF cm^−2^)	*P*_(EIS)_%
0.00	2.74	50	183	—
50	1.74	170	151	70.58
150	1.68	263	143	80.98
250	1.63	344	133	85.46
350	1.66	571	128	91.24
450	1.52	964	119	94.81

From the fitting equivalent circuit the double layer capacitance *C*_dl_ were determined from eq.

7 *C*_dl_ = *Y*_0_(*W*_max_)^*n*−1^

where, *w* is the angular frequency, *Y*_0_ and *n* are, respectively, the values of the CPE admittance and exponent.8
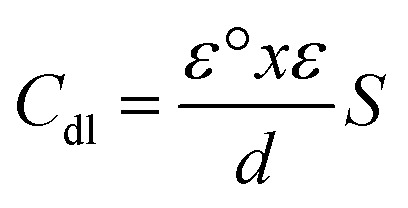
Where *d*, is the adsorbed film thickness, *ε*° is the permittivity of air of the medium *ε* (dielectric constant) and *S* is the electrode surface area.

The decrease in the values of *C*_dl_ with the increase of the thickness of the adsorbed layer gives evidence to the protective film formed on the X80 CS surface.^37^ For more declaration of the protective action of the parsley oil on the corrosion process, the Bode plots are represented in Fig. 2b as shown, the impedance modulus, has an increase with the increase of the parsley oil at low frequencies, and hence proving the adsorption of this compound in the X80 CS surface and in turn improving the inhibitory action against H_2_SO_4_ solution.^37^ Also, the appearance of a single peak in the phase angle plots that demonstrates the occurrence of a single time constant at the steel/solution interface.

### 3.3. Parsley oil as pitting inhibitors

**Fig. 4:**
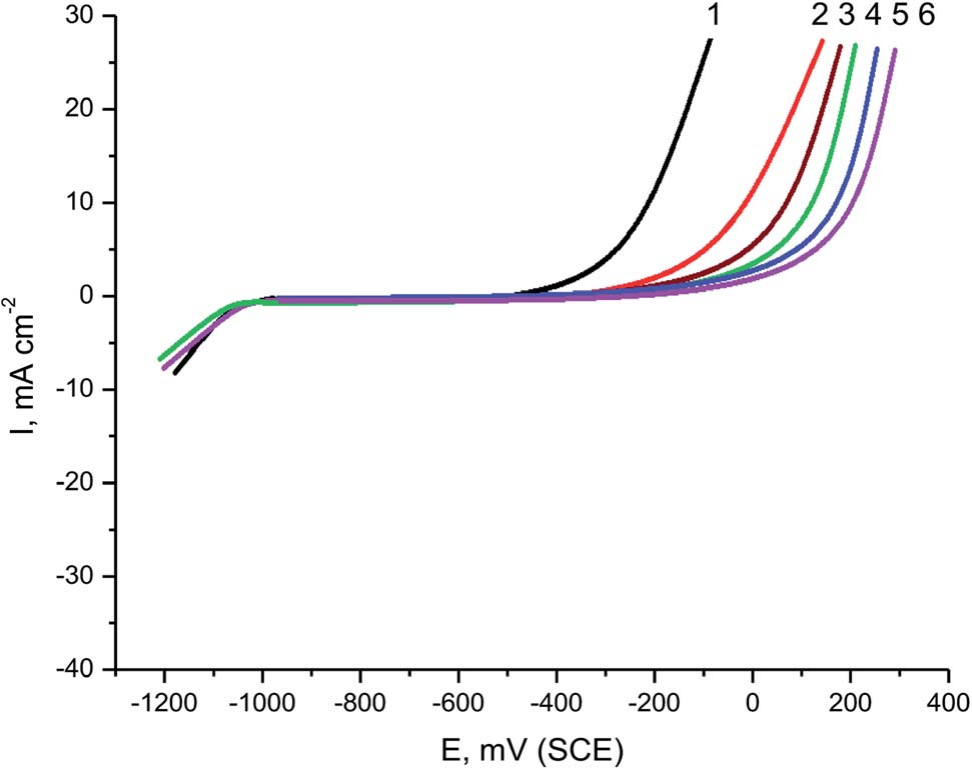
PDAP curves of X80 CS in 0.5 M H_2_SO_4_ + 0.5 M NaCl solutions containing different concentrations of parsley oil. (1) 0.00 (2) 50 (3) 150 (4) 250 (5) 350 (6) 450 ppm.

Parsley oil was tested as a pitting inhibitor for the corrosion of X80 CS electrode in 0.5 M H_2_SO_4_ solution containing chloride ions. Fig. 4 displays the PDAP curves for the X80 CS electrode in a 0.5 M H_2_SO_4_ + 0.5 M NaCl solution including a some doses of parsley oil at a scanning rate of 0.5 mV sec^−1^. Choose a low scan rate to start pitting at the lowest positive potential.^38^ It is evident from this figure that, with increasing potential there is no change of current density, and the dissolution peak is not appeared in the anodic scan. This elucidates the vigor of the oxide layer formed on the steel surface.^39^ After stabilizing the current in the anodic scan until a definite potential is reached, the current rises speedily due to the chloride ions destroying the oxide layer and initiating the pitting aggression. This potential is called pitting potential (*E*_pit._). The effect of adding a different concentration of parsley oil led increased the current stability in the anodic scan and shifted the values of E_pit_ to a more positive direction consistent with this equation:^40,41^

9 *E*_pit_ = *n* + *m* log *C*_oil_

where, *n*, *m* are constants that relied on the type of additive and halide ions used as well as the chemical composition of the carbon steel. The noble shift of the *E*_pit_ values gives an idea for the strength of pitting corrosion.

**Fig. 5:**
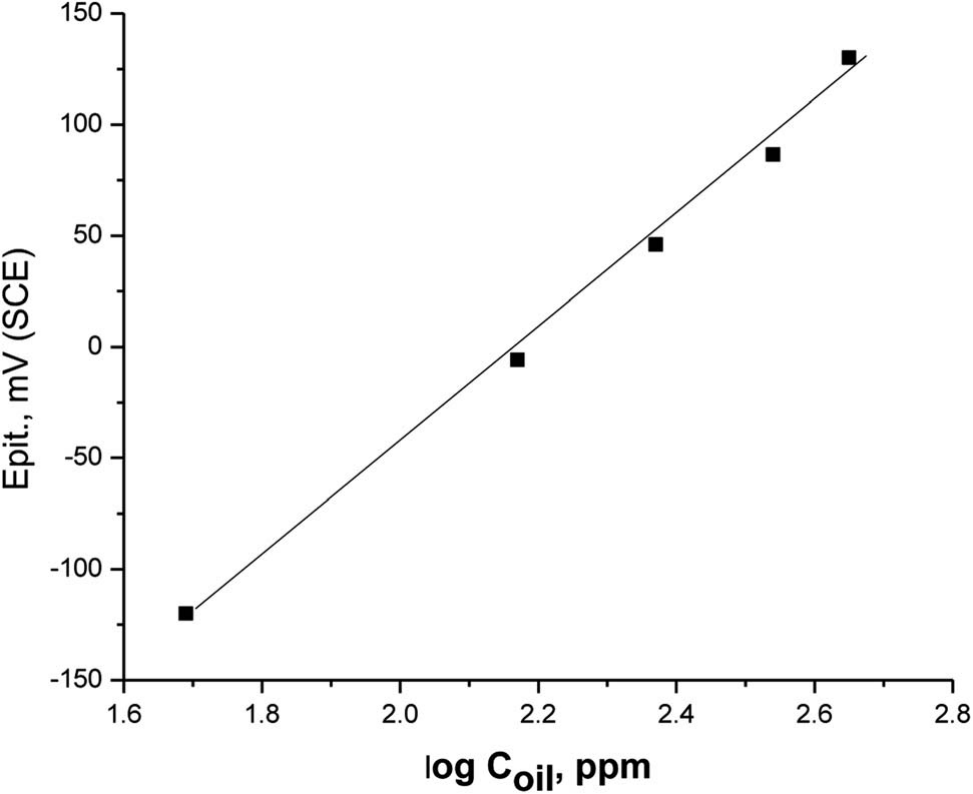
A plot between *E*_pit_ and log *C*_oil_ for X80 CS in 0.5 M H_2_SO_4_ + 0.5 M NaCl solutions_+ different doses of parsley oil.

Fig. 5 displays the relationship between the values of *E*_pit_ and the logarithm of concentration of parsley oil. A straight line was acquired clarifying that with the increase in the concentricity of the parsley oil, the stability of passive film increases and the values of *E*_pit_ turns into a nobler trend. This elucidates that the resistance of the X80 CS to pitting attack is increased and the parsley oil is considered as pitting corrosion inhibitor.

### 3.4. Weight reduction (WR) technique

#### 3.4.1. Influence of parsley oil concentration

The influence of different concentrations of parsley oil ranged from 50 to 450 ppm on the corrosion of X80 CS in 0.5 M H_2_SO_4_ solution was inspected using WR measurements. WR of X80 CS in mg was computed after fixed time 12 hours in the devoid of and including different concentrations of parsley oil. The rate of corrosion ( *K*_corr._) (mg cm^−2^ h^−1^) was computed from the next relation:10
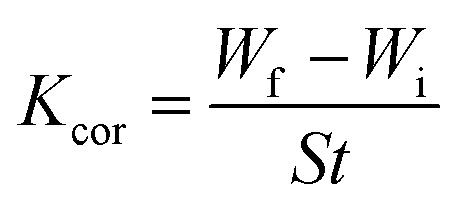
*W*_f_ and *W*_i_ are the WR in the blank 0.5 M H_2_SO_4_ solution and when including the inhibitor, *S* is the surface area and *t* is the time in hours.

The inhibitory efficacy (*P*_wr_%) from WR method and surface coverage (*Θ*) was determined from the next equations and11
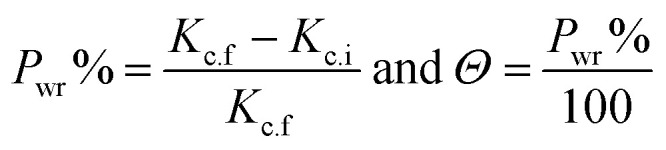
where *K*_c.f_ and *K*_c.i_ are the corrosion rates in the blank 0.5 free 0.5 M H_2_SO_4_ and in the existence of parsley oil.

**Fig. 6:**
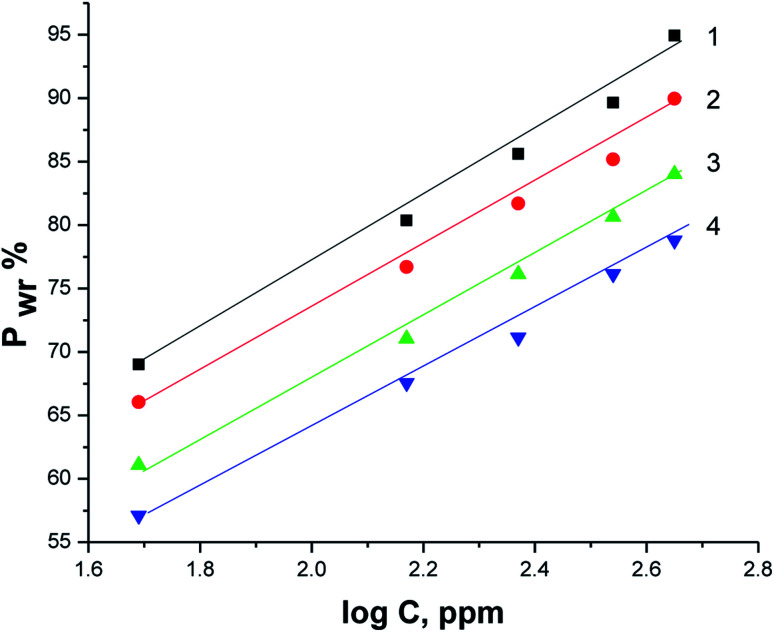
The relationship between *P*_wr_% and log *C*_oil_ for X80 CS in 0.5 M H_2_SO_4_ containing different doses of parsley oil at different temperatures. (1) 298 K (2) 308 K (3) 318 K (4) 328 K.

Fig. 6 illustrates the relationship between the concentration of parsley oil and inhibition efficacy ( *P*_WR_%)at various temperatures. It is evident that with the augmentation the concentration of parsley oil the WR of steel declines and the *P*_WR_% increases. These outcomes demonstrate the inhibitory effect of parsley oil due to its adsorption to the surface of X80CS. The X80CS surface is separated from the corrosive free 0.5 M H_2_SO_4_ by creating of adsorbed film on its surface.^42^

#### 3.4.2. Effect of temperature and computation of the activation parameters

The effect of increasing the temperature on the inhibiting efficacy of parsley oil (*P*_wr_%) onto the dissolution of X80 CS in 0.5 M H_2_SO_4_ solution at different temperatures was inspected using WR measurements. The corrosion parameters are registered in Table 4. The results show that with increasing temperature, the difference in WR and *K*_corr_ values increases while *P*_wr_% and *Θ* values are reduced. This means that the desorption rate of parsley oil is increased on the surface of X80 CS which confirms the physical adsorption of parsley oil. The activation energy (*E*_a_*), the enthalpy of activation (ΔH*)and the entropy of activation (ΔS*) for dissolution of X80 CS in 0.5 M H_2_SO_4_ solution as well as when it contains certain doses of parsley oil was investigated from Arrhenius-equation the alternative Arrhenius relation.^43,44^

12 *K*_corr._ = *A*e^(−*E*_a_*/*RT*)^

13
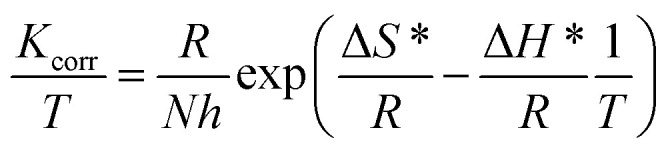
where, *R*, A, *h* and *N* are the gas constant, frequency factor, the Plank's constant and the Avogadro's number, respectively.

**Table 4:** Corrosion parameters acquired from WR measurements

Temperature °K	Conc. of oil (ppm)	WR (mg)	*K*_corr._ × 10^−4^ mg cm^−2^ h^−1^	*Θ*	*P*_(WR)_%
298	0.00	0.328	11.156	—	—
	50	0.098	3.456	0.690	69.02
	150	0.065	2.293	0.794	79.44
	250	0.049	1.728	0.845	84.51
	350	0.033	1.164	0.897	89.65
	450	0.016	0.564	0.949	94.94
308	0.00	0.418	14.744	—	—
	50	0.142	5.008	0.660	66.03
	150	0.104	3.668	0.751	75.12
	250	0.083	2.927	0.801	80.14
	350	0.062	2.186	0.852	85.17
	450	0.042	1.481	0.899	89.95
318	0.00	0.532	18.765	—	—
	50	0.207	7.301	0.611	61.09
	150	0.154	5.432	0.711	71.05
	250	0.127	4.479	0.761	76.13
	350	0.106	3.739	0.801	80.07
	450	0.085	2.998	0.840	84.02
328	0.00	0.641	22.610	—	—
	50	0.275	9.700	0.571	57.09
	150	0.211	7.442	0.671	67.08
	250	0.185	6.525	0.711	71.14
	350	0.153	5.396	0.761	76.13
	450	0.134	4.726	0.791	79.09

**Fig. 7:**
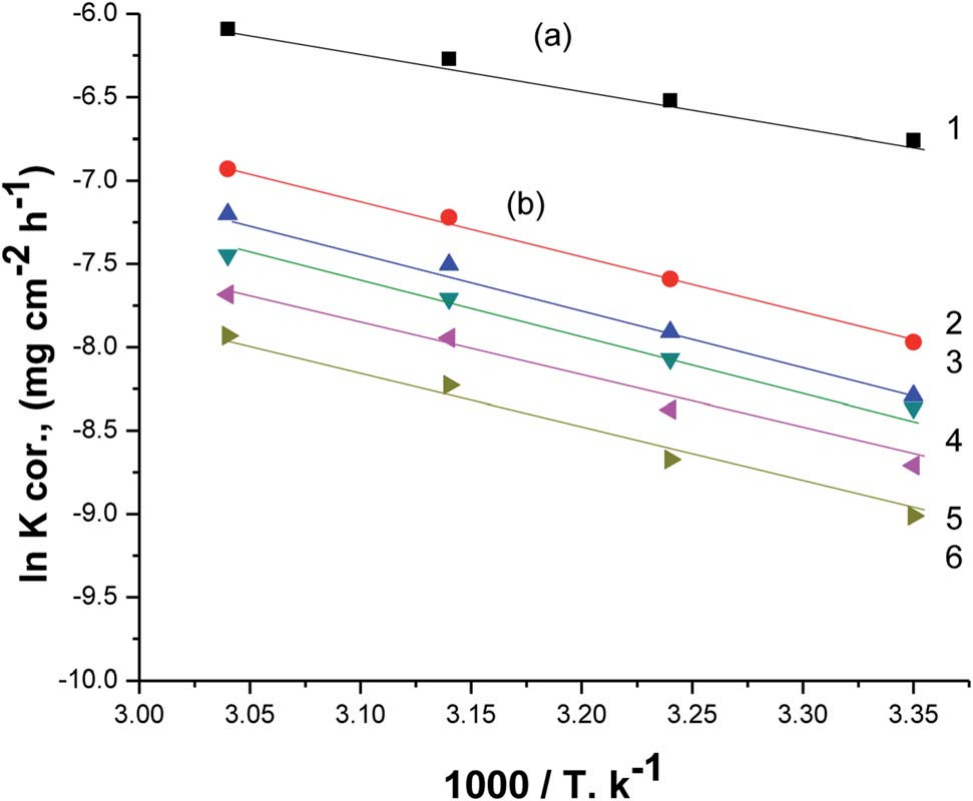
The relation between ln *K*_corr_ and 1/*T* for the corrosion of X80 CS in 0.5 M H_2_SO_4_ including some doses of parsley oil [a] (1) 0.5 M H_2_SO_4_. [b] (2) 50 ppm (3) 150 ppm (4) 250 ppm (5) 350 ppm (6) 450 ppm.

Fig. 7 displays Arrhenius plots (ln *K*_corr_*vs.* 1/*T*) for the dissolution of X80 CS in 0.5 M H_2_SO_4_-free solution and including certain doses of parsley oil. Linear plots were obtained with slope equal (−*E*_a_*/*R*). The *E*_a_* values were computed and registered in Table 5. *E*_a_* was apparently increased by increasing the concentration of parsley oil. This elucidates that the corrosion of X80 CS under these circumstances is controlled by activation. The hike in *E*_a_* values proportional to the concentration and *P*% of parsley oil. This demonstrates that the energy barrier for the dissolution reaction is increased in the existence of parsley oil due to the creation of a adsorbed layer on the surface of the X80 CS. This layer preserves the steel from H_2_SO_4_ solution by preventing the charge/mass transfer interaction that occurs on the X80 CS surface.^45^ When the temperature is increased, the acid molecules get enough heat energy to cross this barrier and further corrosion occur.

**Table 5:** Activation parameters for X80 CS in free 0.5 M H_2_SO_4_ solution and in presence of various concentrations of parsley oil

Conc. of oil (ppm)	*E*_a_ kJ mol^−1^	ΔH* kJ mol^−1^	ΔS* J mol^−1^ K^−1^
0.00	18.29	16.62	447.38
50	28.76	24.95	455.95
150	37.90	34.93	467.54
250	40.23	36.42	475.72
350	48.02	44.57	488.93
450	54.35	51.70	497.64

**Fig. 8:**
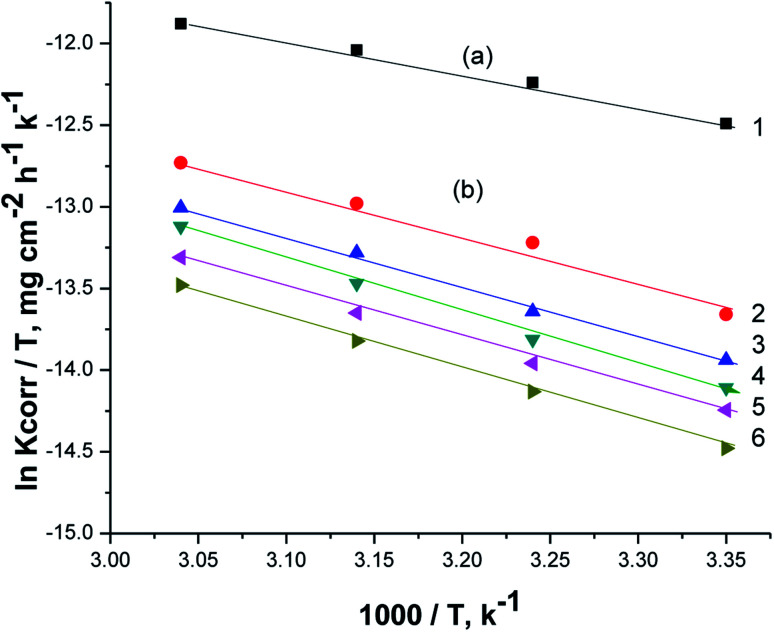
The relation between ln *K*_corr_/*T* and 1/*T* for the corrosion of X80 CS in 0.5 M H_2_SO_4_ including some doses of parsley oil [a] (1) 0.5 M H_2_SO_4_. [b] (2) 50 ppm (3) 150 ppm (4) 250 ppm (5) 350 ppm (6) 450 ppm.

Fig. 8 illustrates the relationship between (ln *K*_corr._/*T vs.* 1/*T*) for the corrosion of X80 CS in free 0.5 M H_2_SO_4_ solution and containing some doses of parsley oil. A linear lines were obtained. The ΔH* and ΔS* values were determined from the slope and intercept, respectively, and registered in Table 5. The positive signs of ΔH* and elevated with increase of parsley oil concentration which confirm the endothermic nature of dissolution of X80 CS. This proves that the presence of parsley oil reduces the corrosion of X80 CS in free 0.5 M H_2_SO_4_ solution.

Negative ΔS* values confirm that the rate-limiting step in the activated complex represents binding instead of detachment. This indicates a decrease in disarrangement from the reactants to the activated complex.

### 3.5. Adsorption isotherm and mechanism of inhibition

The basic criteria for any compound to be an effective corrosion inhibitor is its ability to adsorb on the surface of the metal by substituting the water molecules adsorbed on the metal surface and its adsorption to the surface, bearing in mind the surface energy to all sites are equals and the absence of reaction between the adsorbed molecules. In this mode, the number of active sites subjected to acidic solutions is reduced and result in a lower corrosion rate. The adsorption activity relies on various factors such the chemical compositions of the additives used and the existence of some adsorption centers in them, type of metal used, temperature, type and concentration of corrosive media.

A number of mathematical relationships for the adsorption isotherms including Langmuir, Freundlich, Frumkin, Temkin were applied to the data obtained from WR measurements to choose the preferable isotherm for adding different concentrations of parsley oil on X80 CS corrosion of in free 0.5 M H_2_SO_4_ solution.^46^

We find that a suitable isotherm that matches the current data is the Langmuir isotherm according to the subsequent equation:14
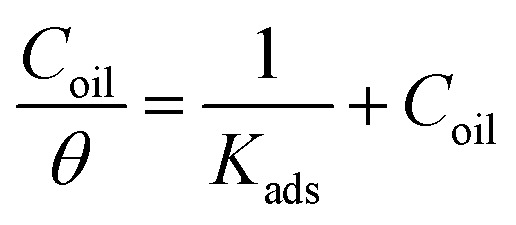
where *C*_oil_ is the concentration of parsley oil, *K*_ads_ is the equilibrium constant of adsorption.

**Fig. 9:**
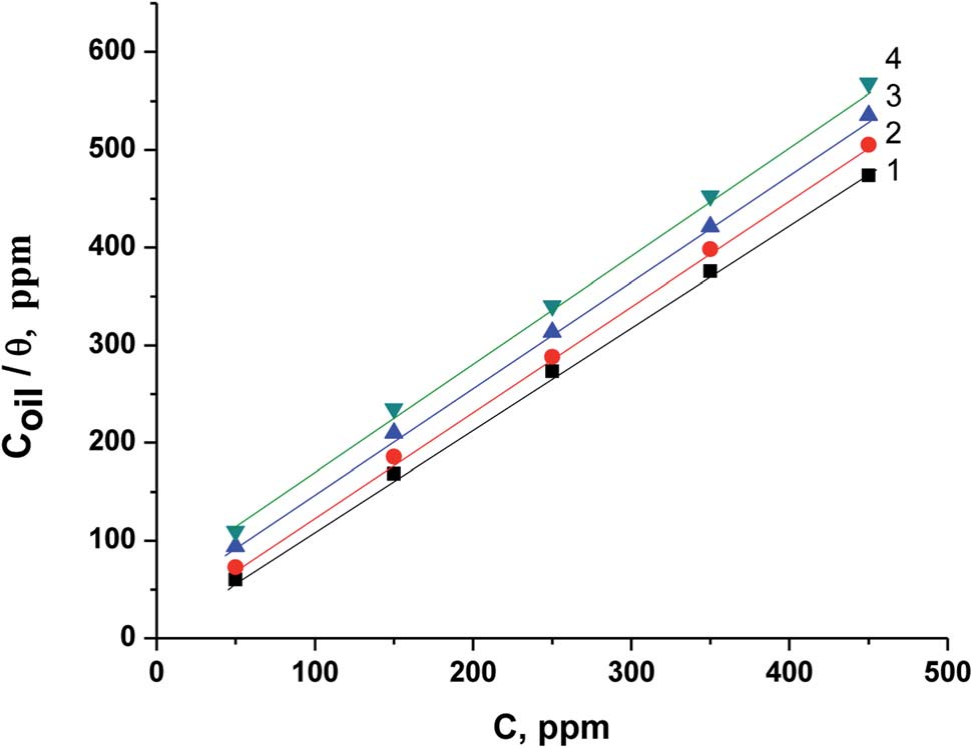
Langmuir isotherm relationship for the corrosion of X80 CS in 0.5 M H_2_SO_4_ including some doses of parsley oil at certain temperatures. (1) 298 K (2) 308 K (3) 318 K (4) 328 K.

Fig. 9 represents the relation between *C*_oil_/*θ* and *C*_oil_ for the corrosion of X80 CS in 0.5 M H_2_SO_4_-free solution and incorporating various concentrations of parsley oil at various temperatures. A straight lines were obtained with slope approximately equal one. The correlation coefficient is equal 0.998. This elucidates the adsorption of parsley oil on the surface of X80 CS according to Langmuir isotherm which means there is zero interaction between the adsorbed species.^47^ The *K*_ads_ values were determined from the intercept of Langmuir plots and equal to 28.5, 21.6, 16.2 and 12.5 ×10^3^ at 298, 308, 318 and 328, respectively. The *K*_ads_ values rise at low temperature and decline with increasing temperature. This indicates a greater tendency to adsorb the parsley oil components on the steel surface by increasing the surface coverage on the X80 CS surface and reducing the corrosion rate.

The free energy of adsorption (Δ*G*_ads._) was comuted from the next equation:15
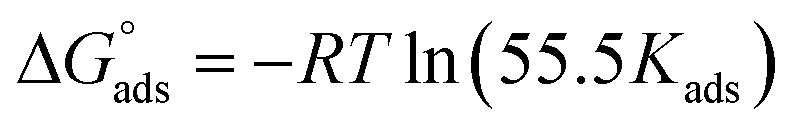


The determined values of Δ*G*_ads._ equal to −36.53, −36.72, −37.52, and −37.98 kJ mol^−1^ at the desired temperatures of at 298, 308, 318 and 328, respectively. The negative marks of Δ*G*_ads._ elucidates that the adsorption of parsley oil on the X80 CS surface are spontaneous. It is evident that from literature^48^ when the Δ*G*_ads._ values are lower than −40 kJ mol^−1^ the adsorption of the inhibitor on the metal surface is chemical while when it is above −20 kJ mol^−1^ the adsorption of inhibitor molecule is physical. So, the computed values of Δ*G*_ads._ ranged between−36.53 and −37.98 kJ mol^−1^. The data confirm that the adsorption of parsley oil on the surface of X80 CS is a mixture between physical and chemical adsorption.

The values of enthalpy of adsorption Δ*H*_ads_ can be computed from the Langmuir equation by applying this equation:^49^16
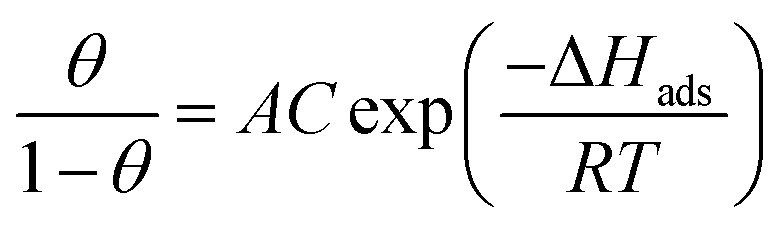
where *C* is the concentricity of parsley oil, *A* is constant.

**Fig. 10:**
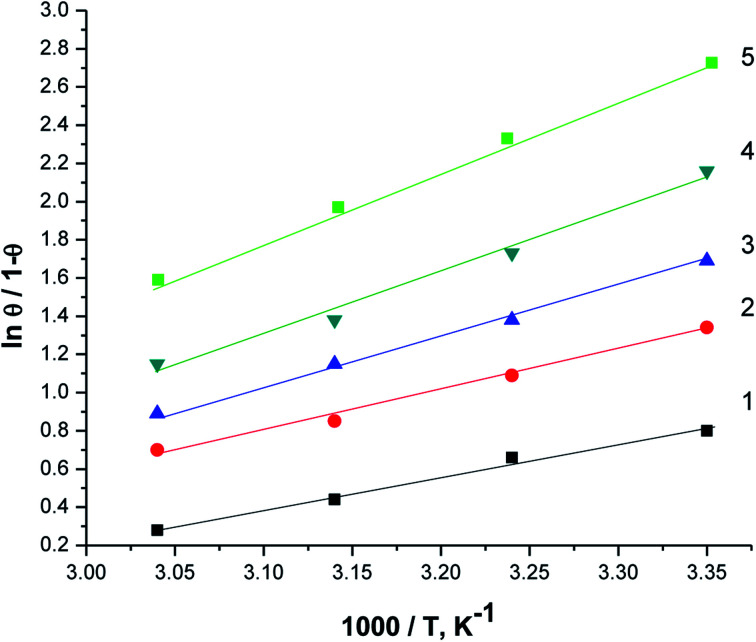
The relationship between log (*θ*/1 − *θ*) *versus* 1/*T* for X80 CS in 0.5 M H_2_SO_4_ containing different doses of parsley oil. (1) 50 ppm parsley oil (2) 150 ppm (3) 250 ppm (4) 350 ppm (5) 450 ppm.

Fig. 10 displays the relation between [log(*θ*/1 − *θ*) *versus* 1/*T*]. The values of Δ*H*_ads_ are computed from the slope of this relations and equal to −22.28, −25.62, −28.67, −29.93, and −32.22 at 50, 150, 250, 350 and 450 ppm of parsley oil, respectively. The negative values of Δ*H*_ads_ demonstrates that the adsorption of parsley oil on the surface of X80 CS is exothermic in nature. This behavior is explained by the fact that the high temperature leads to the removal some of the absorbent layer of parsley oil from the surface of X80 CS.

The entropy of adsorption (Δ*S*_ads_) is calculated from the subsequent equation:*T*Δ*S*_ads_ + Δ*H*_ads_ = Δ*G*_ads_

The determined values of Δ*S*_ads_ are equal to −197, −202, −208 and −207 J mol^−1^ at the temperature 298, 308, 318 and 328 K, respectively. The negative values of Δ*S*_ads_ demonstrate that the randomness decreases when moving from the reactant to the adsorbed surface. This indicates the adsorption strength of the parsley oil on the surface of X80 CS. The values of adsorption thermodynamic functions agree with the reduced values of inhibition efficacy at higher temperatures.

### 3.6. DFT investigation

**Fig. 11:**
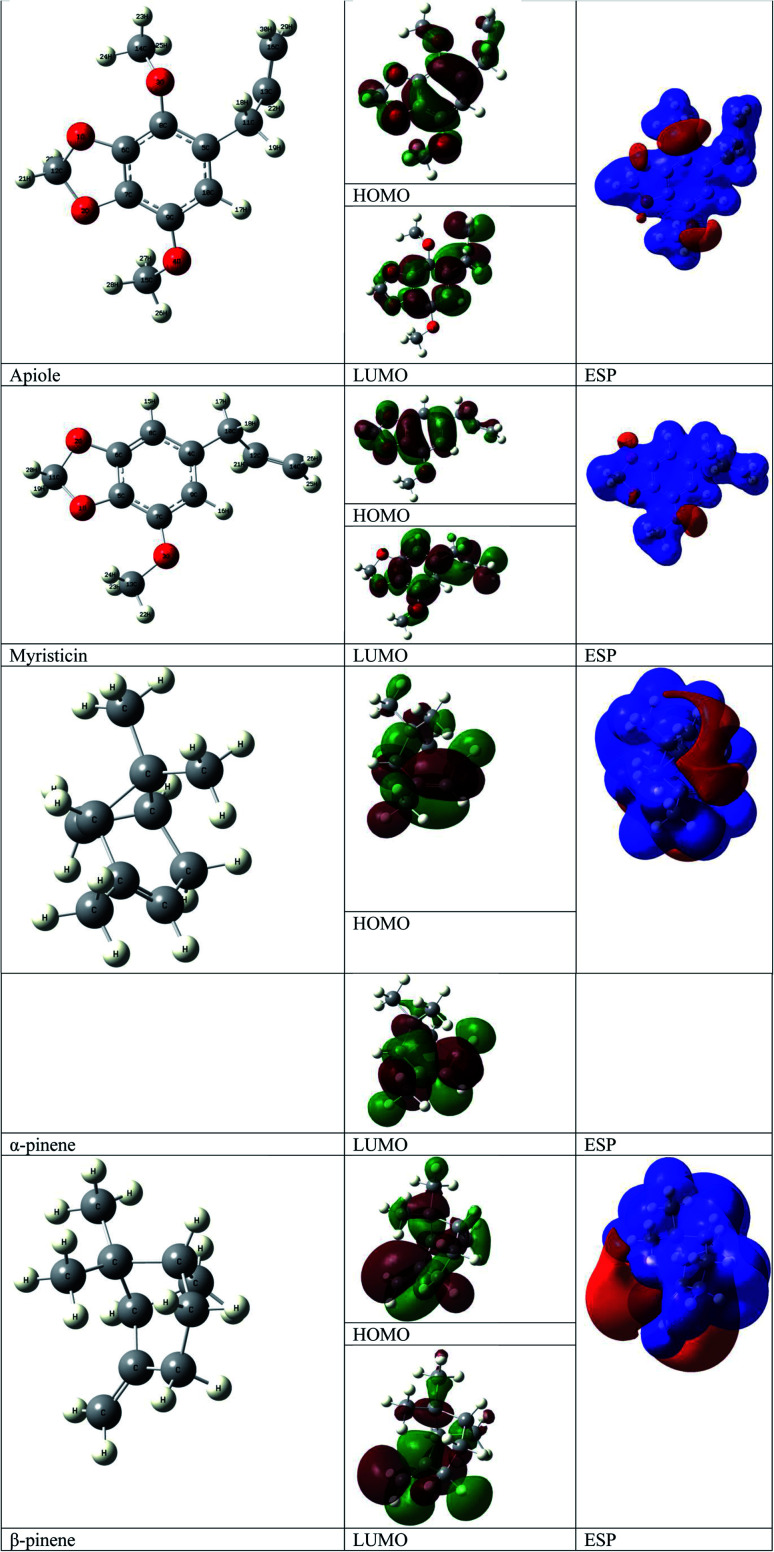
Optimized structure, frontier molecular orbital, and ESP for Apiole, Myristicin, α-pinene and β-pinene molecules.

Natural parsley oil contains four chemical compounds, namely: Apiole, Myristicin, α-pinene, and β-pinene. In a previous study, quantum chemical calculations were performed on both α-pinene and β-pinene compounds based on the density functional theory (DFT).^16^ There are two main compounds called Apiole and Myristicin that are found in natural parsley oil. The Apiole and Myristicin compounds were studied by applying the B3LYP level of theory and 6-31g(d,p) basis set. The goal of theoretical calculations is to compare the four compounds through the electronic structures and predict which molecule gives the highest inhibition efficiency. The optimized structures, frontier molecular orbitals (HOMO, and LUMO), and electrostatic potential maps (ESP) of Apiole and Myristicin compounds were shown in Fig. 11. The HOMO energy level relates to a molecule's capacity to donate electrons to the metal surface's vacant d-orbital, whereas the LUMO energy level refers to the molecule's ability to accept electrons.^50^ With increasing HOMO and decreasing LUMO energy levels, the inhibitor molecule's interaction with the metal surface increases. As seen in Table 6 the values of HOMO and LUMO of the four compounds in gaseous and aqueous phases, the results we obtained show that the Apiole molecule has the largest HOMO value in the gas phase and the lowest value of LUMO energy in both phases and this indicates that Apiole molecule predicts to give the largest inhibition efficiency.

**Table 6:** Quantum parameters of the four compounds found in natural parsley oil

	Gas phase				Aqueous phase			
	Apiole	Myristicin	α-Pinene^a^	β-Pinene^a^	Apiole	Myristicin	α-Pinene^a^	β-Pinene^a^
E_HOMO_ (eV)	−5.48	−5.50	−5.94	−6.23	−5.70	−5.64	−5.97	−6.32
E_LUMO_ (eV)	0.12	0.26	0.80	0.78	−0.05	0.09	0.77	0.68
Δ*E* (eV)	5.60	5.76	6.74	7.01	5.65	5.73	6.74	7.00
*μ* (Debye)	2.74	1.56	—	—	3.66	2.14	—	—
*η* (eV)	2.80	2.88	3.37	3.51	2.83	2.87	3.37	3.50
*σ* (eV)^−1^	0.357	0.347	0.297	0.285	0.354	0.349	0.297	0.286
Δ*N* (e)	0.38	0.38	0.33	0.30	0.34	0.36	0.33	0.29

a Ref. 16.

Fig. 11, shows the frontier molecular orbitals distribution, the HOMO and LUMO distributions for the Apiole and Myristicin molecules are characterized by inequality. These results indicate that both Apiole and Myristicin molecules possess active sites which interact with the metal surface.^51^

The energy gap (Δ*E*) is a useful quantum parameter that measures the stability and reactivity of the molecule. As the energy gap of the components of parsley oil decreases, the effectiveness of the parsley oil increases towards adsorption on the X80 CS surface leading to an increase in the inhibition efficacy.^52,53^ As seen in Table 5, the Apiole molecule has the lowest energy gap in both gas and aqueous phases, therefore the Apiole molecule easily adsorbs on the X80 CS surface.

Chemical hardness (*η*) refers to a chemical species' resistance to electron cloud polarisation or deformation. The inhibition efficiency is inversely related to the chemical hardness of a compound. The energy gap, chemical hardness, and softness (*σ*) are all related to each other. Chemical hardness and softness are based on HOMO and LUMO energy values as a result of HSAB's theorem, as is widely known. Soft molecules with a small HOMO–LUMO energy gap can be employed as corrosion inhibitors because they have a low HOMO–LUMO energy gap. As presented in Table 5, in gas and aqueous phases, the Apiole molecule has the highest softness and lowest hardness that emphasizes the Apiole molecule has the highest inhibition efficiency.

Dipole moment (*μ*) is caused by the irregular distribution of charges on the different atoms in the molecule. The dipole moment is directly proportional to inhibition efficiency.^54^ The compound that has the highest *μ* gives the largest inhibition efficiency. As tabulated in Table 5, the Apiole molecule has the highest dipole moment in the gas and aqueous phase.

The parameters obtained from quantum chemical calculations indicate that replacing a hydrogen atom in the Myristicin molecule with a methoxy group has led to a reduction in the energy gap and an increase in the dipole moment. Therefore, the Apiole molecule gives higher efficiency than the Myristicin molecule.

The fraction of the electron transferred (Δ*N*) is calculated for the Apiole and Myristicin molecules and given in Table 5. According to Lukovits study,^55^ the values of Δ*N* <3.6, the ability of the parsley oil to donate an electron to the X80 CS surface increase, and the inhibition efficiency increases. The results obtained for Apiole and Myristicin molecules are well adsorbed on the Fe(110) surface.

Electrostatic potential maps (ESP) are a visual tool that relates to the electrophilic and nucleophilic sites in a molecule. The blue and red colors refer to the positive and negative regions respectively. As seen in Fig. 11, the negative charge is distributed on the oxygen atoms in both Apiole and Myristicin molecules that reveal the reactive sites of a molecule. In α-pinene and β-pinene, the negative potential surrounds methyl and methylene groups.

**Fig. 12:**
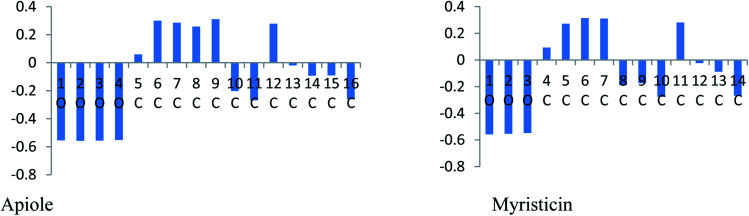
Mulliken charges for Apiole and Myristicin molecules.

**Fig. 13:**
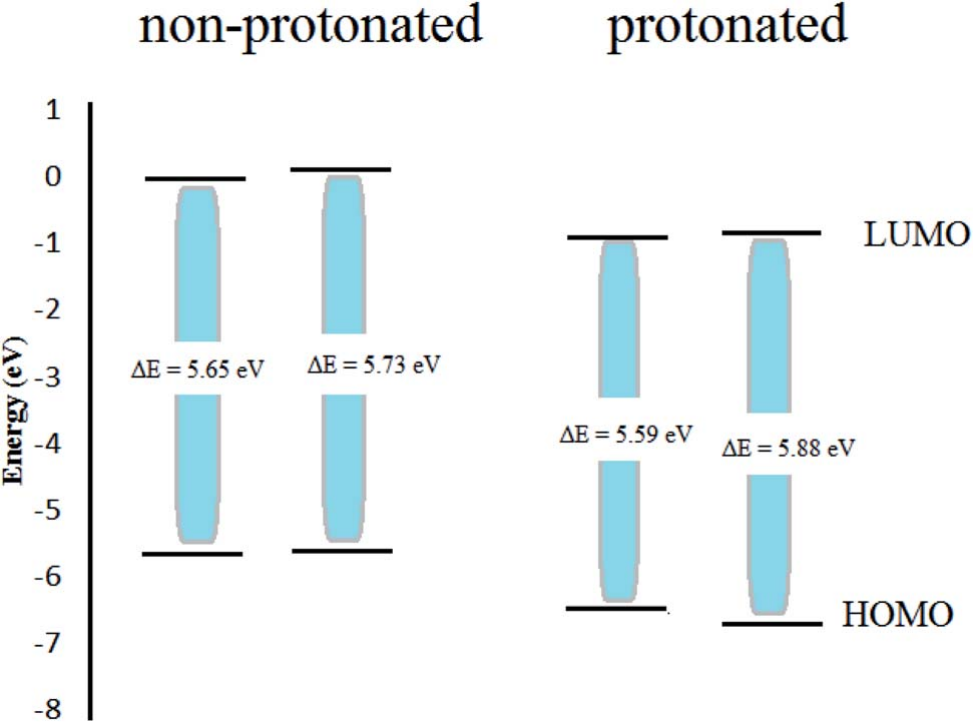
The unprotonated and protonated forms for Apiole and Myristicin molecules.

In an acidic solution, the Apiole and Myristicin molecules can be protonated through an oxygen atom (O2 for Apiole and O1 for Myristicin molecules) that has the highest negative charge as seen in Fig. 12, that obtained from the Mulliken population analysis. In the comparison between the protonated and non-protonated forms as seen in Fig. 13, through *E*_HOMO_, *E*_LUMO_, and Δ*E*. we found that the protonated form *E*_HOMO_ is lower than the non-protonated form, implying that the non-protonated form has a more tendency to donate electrons to metal atoms than the protonated form. The protonated form of *E*_LUMO_ exhibits lower values than the non-protonated form, indicating that the protonated form has a higher ability to accept electrons from the metal than the non-protonated form. For the Apiole molecule, the non-protonated form has a greater energy gap (Δ*E*) than the protonated form, indicating that the protonated form has a greater ability to adsorb on the metal surface.

### 3.7. MC simulation

**Fig. 14:**
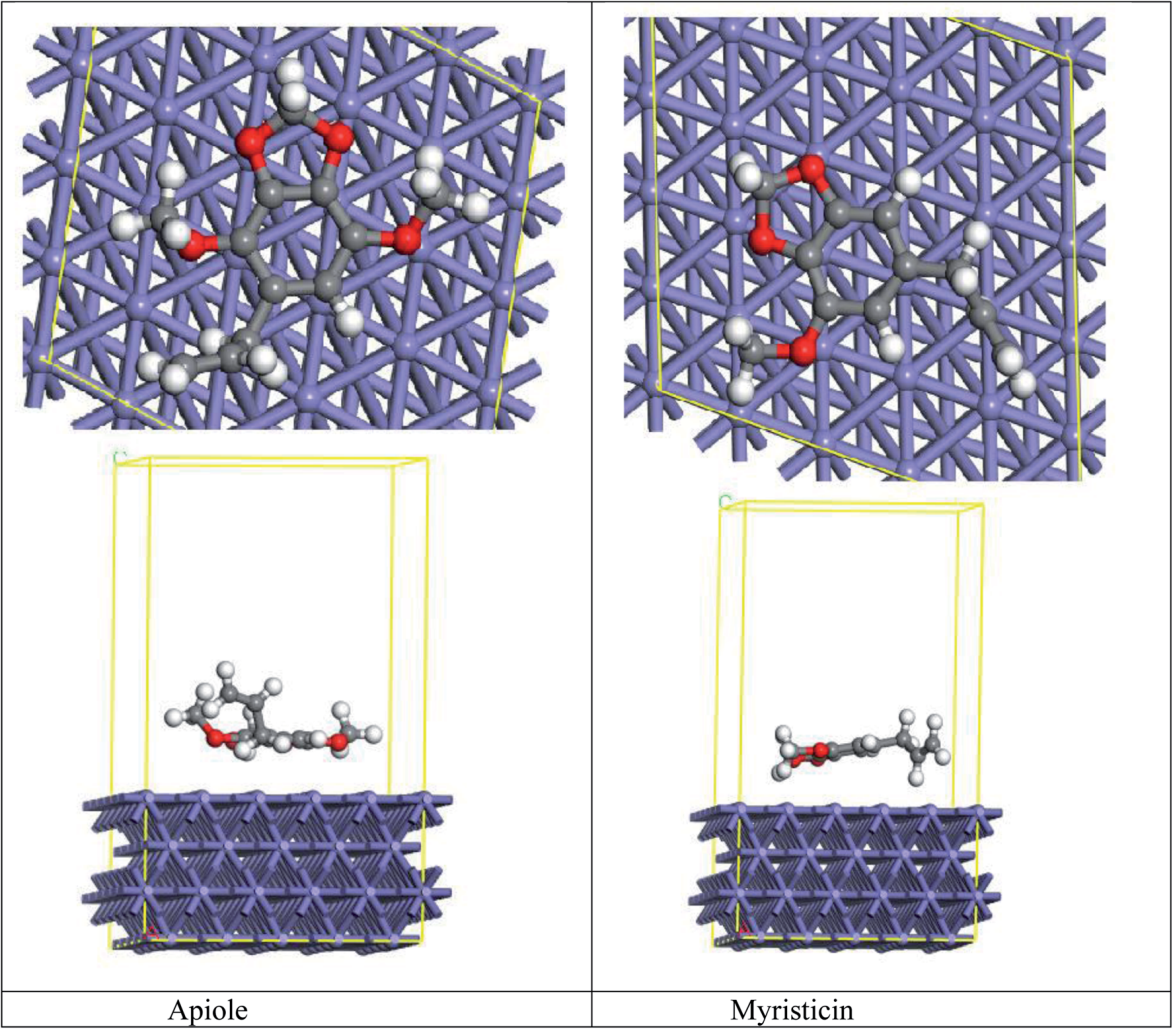
Top and side view for adsorption of Apiole and Myristicin molecules on Fe(110).

MC simulation is a tremendous tool that is used to determine the adsorption of the Apiole and Myristicin molecules on the Fe (110) surface. Both Apiole and Myristicin molecules were simulated in the gas phase. As seen in Fig. 14, which shows that the two inhibitors adsorbed on Fe (110) in a parallel adsorption configuration, and Table 7 show the adsorption energy of the inhibitors on Fe (110). The higher adsorption energy of Apiole inhibitor than Myristicin inhibitor leads to the strong interaction of Apiole inhibitor on Fe (110) which shows higher inhibition efficiency.

**Table 7:** The binding energy of the four components present in natural parsley oil

System	Interaction energy (kcal mol^−1^)	Binding energy (kcal mol^−1^)
Fe(110) + Apiole	−112.92	112.92
Fe(110) + Myristicin	−98.25	98.25
Fe(110) + α-pinene^a^	−72.18	72.18
Fe(110) + β-pinene^a^	−69.15	69.15

a Ref. 16.

## 4. Conclusion

Parsley oil acts as an excellent effective inhibitor for the corrosion of X80 CS in 0.5 M H_2_SO_4_ using electrochemical and chemical methods and the outcomes have been correlated with the calculations of theoretical studies. Galvanostatic polarization indicated that the parsley oil acted as mixed inhibitor with a dominant control of the cathodic reaction. The inhibition impact of the parsley oil owing to its spontaneous adsorption on the X80 CS surface. Adsorption is subject to the Langmuir isotherm. Parsley oil showed good efficacy against pitting corrosion of X80 CS by turning the pitting potential to nobler trend. We investigated the four components of parsley oil based on DFT. The results obtained from the quantum chemical calculation showed that the Apiole molecule gives the highest inhibition efficiency. The adsorption of the parsley oil on the Fe(110) is parallel to the metal surface.

## Conflicts of interest

There are no conflicts to declare.

## Supplementary Material
